# Trajectories of Patient-Reported Outcomes among Diverse Cancer Patients in Ambulatory Oncology Clinics

**DOI:** 10.21203/rs.3.rs-7810530/v1

**Published:** 2025-10-26

**Authors:** Akina Natori, Blanca Silvia Noriega Esquives, Vandana D. Sookdeo, Rui Gong, Jessica MacIntyre, Patricia I. Moreno, Carmen Calfa, Tracy E. Crane, Matthew Schlumbrecht, Frank J. Penedo

**Affiliations:** University of Miami; Sylvester Comprehensive Cancer Center; Sylvester Comprehensive Cancer Center; University of Miami; Sylvester Comprehensive Cancer Center; University of Miami; University of Miami; University of Miami; University of Miami; University of Miami

**Keywords:** Cancer Survivors, Patient-Reported Outcome Measures, Health-Related Quality of Life, Longitudinal Studies

## Abstract

**Purpose:**

Patient-reported outcomes (PROs) offer critical insights into cancer survivors’ symptoms and health-related quality of life (HRQOL). This study evaluated longitudinal trajectories of PROs among cancer survivors across diverse cancer types and identified factors associated with symptom burden and HRQOL.

**Methods:**

We conducted a retrospective longitudinal observational study of 3,809 cancer survivors in ambulatory oncology clinics who completed electronic health record-integrated PRO assessments measuring anxiety, depression, pain interference, fatigue, and physical function, and HRQOL. Linear mixed-effect models evaluated PRO trajectories over time across seven common cancer types.

**Results:**

Survivors with gynecological cancers reported the highest symptom burden across multiple domains, particularly anxiety, pain interference, physical function, and fatigue. Male genital system cancer survivors consistently showed lower symptom burden and better HRQOL. Head and neck cancer survivors improved in pain interference, fatigue, physical function, and HRQOL over time compared to other cancer types, while gastrointestinal cancer survivors exhibited worsening HRQOL than hematologic and head and neck cancer survivors. Marital status and comorbidity burden were independent predictors for all PRO scores.

**Conclusions:**

PRO trajectories varied substantially by cancer types, underscoring the need for personalized, cancer-specific survivorship care strategies.

**Implications for Cancer Survivors:**

While standard follow-up may suffice for male genital system and head and neck cancer survivors, more intensive interventions may be required for gynecological and gastrointestinal cancer survivors to address persistent symptom burden. Routine, EHR-integrated PRO monitoring can identify cancer survivors at risk for persistent symptom burden, guiding timely, tailored interventions to improve long-term HRQOL.

## Introduction

Patient-reported outcomes (PROs) capture patients’ perceptions of their health status, quality of life, and the impact of disease and treatment on their daily lives, without interpretation by healthcare providers [1]. PROs encompass a broad spectrum of self-reported information, including physical symptoms, emotional well-being, functional status, and health-related quality of life (HRQOL)[2]. In clinical research and practice, PROs provide invaluable insights into patients’ experiences, enhancing the understanding of treatment efficacy, guiding clinical decision-making, and promoting patient-centered care [3, 4].Over the past few decades, PROs have been increasingly integrated into clinical trials to assess the effectiveness and tolerability of investigational therapies. More recently, the implementation of PROs in routine clinical workflows, particularly in chronic disease management such as cancer, has gained momentum. PROs are now frequently used in quality improvement initiatives, allowing for continuous symptom monitoring and identifying areas where interventions may improve outcomes [5].

Beyond symptom assessment, PROs, particularly HRQOL measures, are emerging as important prognostic and predictive tools in oncology [6]. Numerous studies have demonstrated that better HRQOL is often associated with better treatment responses and improved survival outcomes in patients with cancer [7–10]. For example, a large-scale analysis of the data from 30 randomized controlled trials across 11 cancer types conducted by the European Organization for Research and Treatment of Cancer (EORTC) found that specific HRQOL domains provided independent prognostic information after adjusting for clinical and sociodemographic variables [11, 12]. Similarly, research from a population-based cancer registry in the Netherlands found a strong association between HRQOL summary scores and overall survival across 12 different cancer types [13].

Despite these findings, the prognostic value of different PRO domains can vary by cancer type, and most research to date has been cross-sectional, limiting understanding of longitudinal trajectories of PROs [8]. Understanding how PROs change over time, particularly across different types of cancer, is crucial for identifying vulnerable patient populations and key domains for targeted interventions. While some longitudinal studies have explored PRO trajectories in specific cancer types or treatment contexts, comprehensive analyses that span multiple cancer types are scarce. Such comparisons are vital for identifying patterns and differences in PRO trajectories that could inform personalized care strategies. This observational longitudinal study aims to identify the trajectories of various PRO domains across multiple cancer types and examine the associations between these trajectories and patient characteristics.

## Methods

### Patient Population

The Sylvester Comprehensive Cancer Center (SCCC) implemented the My Wellness Check (*MWC*) program, a routine electronic health record (EHR)-based PRO and needs screening system, across ambulatory oncology clinics. The *MWC* questionnaire is linked to patients’ scheduled ambulatory oncology clinic visits. If a patient has completed the questionnaire within the past 30 days, it is not re-assigned; otherwise, it is administered again with the next eligible visit. As a result, the timing of PRO assessments is not based on fixed intervals. Patients receive the *MWC* questionnaire via the patient portal. The *MWC* questionnaire includes five PROMIS^®^ computerized adaptive tests (CATs)—Anxiety, Depression, Pain Interference, Fatigue, and Physical Function—to assess common cancer-related symptoms [14–18]. It also includes the Functional Assessment of Cancer Therapy-General (7-item version; FACT-G7) [19] to evaluate HRQOL. Participation in the *MWC* questionnaire is optional. Detailed program descriptions have been published previously [20, 21].

Among 13,403 patients who were assigned to the *MWC* questionnaire between May 2022 and November 2023, patients aged 18 years or older, diagnosed with cancer at any site, regardless of stage or time since diagnosis, and who completed the *MWC* questionnaire at least three times, were included in this study. The institutional review board approved the study protocol at the University of Miami (ePROST#20200984). Informed consent was waived because this study retrospectively used the clinical data that was routinely collected as a standard of care.

### Data Collection

*MWC* questionnaire scores are automatically calculated and stored in the EHR. For this study, all relevant scores were retrospectively collected from the electronic data warehouse (EDW). The main outcomes of interest were HRQOL, as assessed by the FACT-G7, and symptom burden, as assessed by the five PROMIS^®^-CAT domains in the *MWC* program [14–19]. PROMIS^®^ measures use a T-score metric, where 50 represents the mean of a relevant reference population, and 10 represents the standard deviation. Higher scores indicate an increase in the measured construct (e.g., greater depression, better physical function). In addition, a change of 2 to 6 T-score points is widely accepted as a clinically relevant difference, applicable to both within-person changes and comparisons between distinct groups [22]. FACT-G7 score ranges from 0 to 28, which higher scores indicate better HRQOL [23]. Patient demographics, including age, sex, race/ethnicity, marital status, and insurance status, and clinical characteristics at baseline, including cancer site, time since cancer diagnosis, cancer stage, current treatment type, and comorbidities, were also collected from the EDW. Charlson comorbidity index was calculated to assess the severity of comorbidity [24]. Receipt of treatment was defined as a treatment within 30 days before the first assessment.

### Statistical Data Analysis

Due to the limited number of patients with other cancer types, only the seven most frequent cancer types, including breast, gynecological (GYN), gastrointestinal (GI), head and neck, hematologic, lung, and male genital system (MGS) (e.g. prostate, and testicular) malignancies, were included. Descriptive statistics were calculated for baseline characteristics using chi-square tests and analysis of variance (ANOVA) tests where appropriate.

Linear mixed-effects models were employed to assess the impact of time and cancer type on PROMIS and FACT-G7 scores. These models are particularly useful for analyzing repeated measures data, where assessments (or time points) are nested within individuals [25]. At level 1, time was treated as a continuous variable. Person-level predictors (level 2) included cancer site (reference category = male genital system), age at baseline, marital status (partnered: yes/no), race/ethnicity (reference: non-Hispanic White), insurance status (insured: yes/no); time since diagnosis (in years), the presence of metastatic disease (yes/no), Charlson Comorbidity Index, and treatments performed at baseline (radiotherapy, chemotherapy, surgery, immunotherapy, and hormonal therapy, coded as yes/no). Sex was not included in the models due to the high correlation with cancer types. Continuous variables (i.e. age, comorbidities, and time since diagnosis) were grand mean-centered before modeling. We added the random effects of time and intercept to the model to account for variability in the number of assessments and their intervals, especially considering that the MWC questionnaire is linked to patient clinic visits. Additionally, we included an interaction term for the time of assessment by cancer type. Unstandardized regression coefficients (Bs), standard errors (SE), and *p*-values were assessed. Due to the multiple comparisons, a Bonferroni-adjusted significance level of 0.0083 was calculated to account for the increased possibility of type-I error. The predicted means of individual cancer types were plotted graphically across time. Data analysis was performed using SAS v9.4 (SAS Institute Inc., Cary, NC).

## Results

### Patient and Clinical Characteristics

A total of 4,893 patients completed the *MWC* questionnaire at least three times. After excluding patients with cancer types that did not meet the inclusion criteria, 3,809 patients were included in the analysis. From the first assessment (n = 3,809), patients completed an assessment at an average of 3.4 months (SD = 2.0) for the second assessment (n = 3,809 [100% retention]), 6.2 months (SD = 3.0) for the third assessment (n = 3,809 [100%]), and 8.4 months (SD = 2.9) for the fourth assessment (n = 2,323 [61%]) (Online Resource Tables S1 and S2).

The mean age at baseline (T0) was 64.1 years (SE = 0.2). Of the included patients, 1,734 (45.4%) were female, and 1,617 (42.4%) identified as Hispanic. Most patients were insured (n = 3,758, 98.5%) and partnered (n = 2,461, 64.5%). Hematologic malignancies were the most common cancer type (n = 991, 26%), followed by GI (n = 766, 20.1%) and MGS (n = 678, 17.8%). The average time since diagnosis was 3.5 years (SE = 0.1), and nearly a quarter (n = 815, 21.4%) had metastatic disease at baseline. Additional baseline characteristics, including cancer-specific breakdowns, are summarized in Table 1. Baseline PRO score categories indicate that patients with MGS cancers consistently reported the highest percentages of “normal” levels for anxiety, depression, fatigue, physical function, and HRQOL. Conversely, GYN and lung cancer patients exhibited a higher prevalence of moderate or severe symptoms across most PROs (Table 2). Participants who completed four assessments had higher Charlson Comorbidity Index scores, greater anxiety and fatigue at baseline, and were more likely to have metastatic disease and receive chemotherapy, immunotherapy, or hormonal therapy compared to those who only completed three assessments (Online Resource Table S3).

### Trajectories and Predicted Means of Each Patient-Reported Outcome

**Figure 1:**
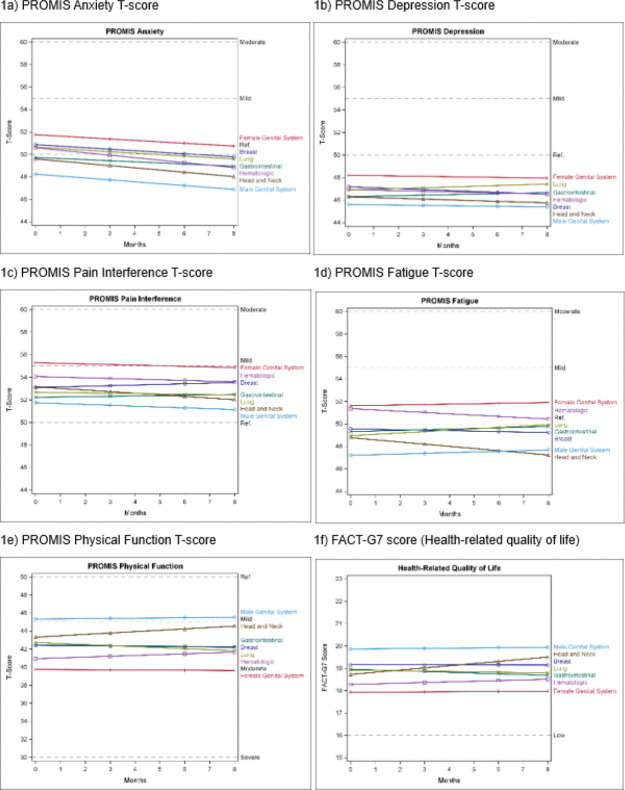
1a-1f Trajectory of PRO by Domain

The final models and results are summarized in Table 3, and the predicted means of each PRO at each time point by cancer type are shown in Online Resource Table S4. Figure 1 illustrates the trajectory of each PRO by cancer type, assuming a random intercept and slope and a reference patient profile (Age: 64.1 years, non-Hispanic White, unpartnered, comorbidities: 6.7, MGS cancer, time since cancer diagnosis: 3.5 years, non-metastatic disease, no treatment at baseline).

### Anxiety

Anxiety scores varied significantly across patients at baseline (random intercept *p* < 0.001). Compared to MGS cancer, patients with all other cancer types except head and neck reported higher anxiety at baseline after adjusting for covariates (all comparisons were significant at p < 0.008). Having a partner (B=−1.4, *p* < 0.001) was associated with lower anxiety, while non-Hispanic Black patients reported lower anxiety than non-Hispanic Whites at baseline (B=−2.4, *p* < 0.001). A greater number of comorbidities was associated with higher baseline anxiety (B = 0.2, *p* = 0.002).

Anxiety scores decreased over time (B=−0.19, p < 0.001) (Fig. 1a). Predicted anxiety scores revealed significantly higher levels for patients with GYN cancer than those with head and neck cancer at all assessment points. Conversely, patients with MGS cancer consistently reported lower anxiety scores than patients with breast, GYN, GI, hematologic, and lung cancers (all comparisons were significant at p < 0.008).

### Depression

Baseline depression scores varied significantly across patients (random intercept *p* < 0.0001). Compared to MGS cancer, patients with GYN and hematologic cancers reported higher anxiety scores at baseline after adjusting for covariates (all comparisons were significant at p < 0.008). In addition, having a partner (B=−1.7, *p* < 0.001) was associated with lower depression, while non-Hispanic Black patients reported lower depression than non-Hispanic Whites at baseline (B=−1.9, *p* < 0.001). A greater number of comorbidities was associated with higher baseline depression (B = 0.2, *p* < 0.001).

Depression scores remained stable over time (B=−0.01, *p* = 0.847) overall. Predicted depression scores revealed significantly lower levels for patients with MGS cancer than patients with GYN cancer at all assessment points (Table S4). Patients with GYN cancer reported significantly higher depression scores than patients with head and neck (at T1 – T4) (all comparisons were significant at p < 0.008).

### Pain interference

Baseline pain interference scores varied significantly across patients (random intercept p < 0.001). Patients with GYN (B = 3.6, *p* < 0.001) and hematologic cancers (B = 2.2, *p* < 0.001) reported significantly higher pain interference scores than those with MGS cancer after adjusting for covariates. In addition, having a partner (B=−1.2, *p* = 0.001) was associated with lower pain interference. Comorbidities (B = 0.4, *p* < 0.001), receiving chemotherapy (B = 0.9, *p* = 0.006), and undergoing surgery (B = 2.2, *p* = 0.002) were significant predictors of higher baseline pain interference scores.

Pain interference scores remained stable over time (B=−0.1, *p* = 0.131). Patients with GYN cancer had higher predicted pain interference scores than those with GI, head and neck, and lung cancers (at T1 – T4). Patients with MGS cancer had lower predicted pain interference scores than those with breast, GYN, and hematologic cancer (at T1 – T4) (all comparisons were significant at p < 0.0083).

### Fatigue

Baseline fatigue scores varied significantly across the patients (random intercept *p* < 0.001). Compared to MGS cancer, patients with breast (B = 2.3, *p* = 0.001), GYN (B = 4.4, *p* < 0.001), GI (B = 2.1, *p* = 0.001), and hematologic cancers (B = 4.1, *p* < 0.001) reported higher fatigue scores at baseline after adjusting for covariates. Having a partner (B=−1.5, *p* < 0.001) was associated with lower fatigue, while Hispanic patients reported lower fatigue than non-Hispanic Whites at baseline (B=−2.4, *p* < 0.001). A greater number of comorbidities (B = 0.4, *p* < 0.001) and receiving chemotherapy (B = 2.3, *p* < 0.001) were associated with higher baseline fatigue.

Fatigue scores remained stable over time (B = 0.1, *p* = 0.027). Compared to patients with head and neck cancer, those with GI (B = 0.2, *p* = 0.002), lung (B = 0.3, *p* < 0.001), and MGS cancers (B = 0.2, *p* < 0.001) reported an increase in fatigue over time. Compared to patients with hematologic cancer, those with GI (B = 0.2, *p* = 0.008), lung (B = 0.3, *p* < 0.001), and MGS cancers (B = 0.2, *p* < 0.002) reported an increase in fatigue over time (Fig. 1d). Patients with MGS cancer had significantly lower predicted fatigue scores than those with GYN, GI, hematologic, and lung cancer at all assessment points (Table S4). Patients with head and neck cancer showed significantly lower predicted fatigue scores than those with GYN and hematologic cancer at all assessment points (all comparisons were significant at p < 0.008).

### Physical Function

Physical function scores varied significantly across patients at baseline (random intercept *p* < 0.001). Compared to patients with MGS cancer, patients with all other cancer types reported lower physical function scores at baseline after adjusting for covariates (all comparisons were significant at p < 0.008). Additionally, older age (B=−0.2), a higher number of comorbidities (B=−0.6), receiving chemotherapy (B=−2.5), and undergoing surgery (B=−2.2) were associated with significantly lower physical function scores (all *p*’s ≤ 0.001). Conversely, having a partner (B = 1.3, *p* < 0.001) and having a longer time since cancer diagnosis (B = 0.1, *p* = 0.004) were associated with significantly higher physical function scores.

Physical function scores remained stable over time (B = 0.04, *p* = 0.735). However, patients with head and neck cancer reported significant improvements in physical functioning over time compared to those with breast (B = 0.2, *p* = 0.008), GI (B = 0.2, *p* = 0.004), and lung cancers (B = 0.3, *p* < 0.001) (Fig. 1e). Furthermore, patients with lung cancer reported a significant decline in physical functioning over time compared to those with hematologic cancer (B = 0.2, *p* = 0.003). Patients with MGS cancer exhibited significantly higher physical function scores compared to those with other cancer types at all assessment points (Table S4). Similarly, patients with head and neck cancer had significantly higher physical function scores compared to those with breast, hematologic, and lung cancer (at T2-T4). In contrast, patients with GYN cancer consistently exhibited lower physical function scores than patients with breast, GI, and head and neck cancer (all comparisons were significant at p < 0.008).

### HRQOL

HRQOL scores varied significantly across patients at baseline (random intercept *p* < 0.001). Compared to patients with MGS cancer, those with GYN (B=−2.0, p < 0.001), GI (B=−0.9, p = 0.003), head and neck (B=−1.1, p = 0.001), and hematologic (B=−1.5, p < 0.001) malignancies had significantly lower HRQOL scores at baseline after adjusting for covariates. A higher number of comorbidities (B=−0.2) and receiving chemotherapy (B=−1.1) were associated with lower HRQOL scores at baseline (*p*’s < 0.001), whereas having a partner (B = 0.9, *p* < 0.001) and having a longer time since diagnosis (B = 0.1, *p* < 0.001) were associated with higher HRQOL scores.

HRQOL scores remained stable over time (B = 0.01, *p* = 0.761). However, patients with head and neck cancer reported significant improvements in HRQOL over time compared to those with GI (B = 0.1, p < 0.001) and lung cancers (B = 0.1, p = 0.003) (Fig. 1f). Compared to patients with GI cancer, those with hematologic cancer reported significant improvements in HRQOL over time (B = 0.1, p < 0.001). MGS cancer patients had higher predicted HRQOL scores than those with breast, GYN, GI, hematologic, and lung cancer at all assessment points (Table S4). Similarly, patients with head and neck cancer had significantly higher HRQOL scores compared to those with GYN and hematologic cancer (at T3-T4).

## Discussion

This study provides a comprehensive analysis of PROs among a diverse cohort of cancer survivors receiving care in ambulatory oncology clinics. By examining longitudinal patterns of anxiety, depression, pain interference, fatigue, physical function, and HRQOL, we identified significant differences in symptom burden and HRQOL that persist over time across multiple cancer types. Patients with GYN cancer reported a relatively greater symptom burden, while patients with MGS cancer reported fewer symptoms compared to patients with other cancer types. Patients with head and neck cancer improved in pain interference, fatigue, physical function, and HRQOL over time, whereas patients with GI cancer reported worsening in HRQOL. In addition, marital status and comorbidities were determinants of all PRO scores. These findings highlight the critical need for personalized care strategies to address the unique challenges different cancer populations face.

Our findings align with existing literature that underscores the variability of PROs across different cancer types. For instance, the consistently lower symptom burden and higher HRQOL observed in patients with MGS cancer have been previously reported, reflecting the generally favorable prognosis and lower treatment-related morbidity associated with these malignancies [26, 27]. Conversely, patients with GYN cancer exhibited the highest symptom burden across multiple domains, particularly in anxiety, pain interference, fatigue, and physical function. These findings are consistent with studies suggesting that GYN cancer survivors often reported significant emotional and physical challenges, partly due to the complex nature of their treatments and the impact on reproductive and sexual health [28, 29]. Beyond clinical factors, cultural or social factors may influence how patients report symptoms. For instance, men adhering to traditional masculine norms may hesitate to discuss symptoms due to fear of vulnerability or weakness openly [30]. Additionally, certain symptoms, such as mental health problems, carry a stigma, leading some patients to underreport or avoid discussing them [31, 32]. Understanding each patient’s unique circumstances is essential for effective cancer care.

The worsening trajectories in HRQOL observed in patients with GI cancer are also consistent with previous studies indicating that GI cancers are often associated with high symptom burden and poor prognosis.[33–35] Patients with GI cancers often face symptoms related to eating-related problems, pain interference, fatigue, and emotional distress, contributing to the overall decline in HRQOL over time [36]. Interestingly, our study identified patients with head and neck cancer as a unique group that demonstrated improvement in several PRO domains over time. This contrasts with some prior studies that have reported persistent or worsening symptomatology in this population, potentially reflecting differences in cancer supportive care, successful implementation of survivorship care plans, and access to psychosocial support to address communication difficulties, body image, and coping strategies [37–40].

The significant variability in PRO trajectories by cancer type suggests that a one-size-fits-all approach to symptom management in oncology is insufficient. For example, the stable or improving symptoms observed in patients with MGS and head and neck cancers may indicate that standard follow-up care may be sufficient for these groups. However, the high and persistent symptom burden in patients with GYN and GI cancers highlights the need for more intensive and tailored interventions. These may include integration of multidisciplinary survivorship clinics that can address multiple complex symptoms through coordinated services spanning oncology, psychosocial support, palliative care, and rehabilitation services. Moreover, leveraging PRO data for real-time symptom management is essential. The My Wellness Check program currently uses PROMIS threshold scores to trigger alerts for healthcare providers; such systems should be more widely adopted to guide timely referrals and interventions. Importantly, our findings suggest that threshold scores and response protocols may need to be refined based on cancer type to optimize care. For example, referral pathways or clinical follow-up may be prioritized for cancer types with consistently higher symptom burdens, such as GYN and GI cancers, to reduce avoidable distress and functional decline.

The significant associations between marital status and better PRO scores across all domains underscore the potential role of social support in mitigating symptom burden. Interventions to strengthen social networks, particularly for unpartnered patients, could enhance their overall well-being [41, 42]. For instance, connecting cancer patients with others who have similar experiences can provide a sense of community and emotional support. Furthermore, online support groups can offer a convenient way for patients to connect with others, especially those in rural or isolated areas. We also found that comorbidities were associated with worse PROs at baseline. Managing multiple health conditions can be overwhelming and contribute to increased anxiety, depression, and stress. Additionally, comorbidities can interact with cancer treatments, leading to adverse side effects or complications. Our findings emphasize the importance of managing comorbidities in cancer survivors to improve their quality of life.

This study’s strengths include its large, diverse sample and use of a validated, EHR-integrated PRO system for real-time data collection. Additionally, the consistent use of the same symptom assessment instrument across all time points enhances the reliability of observed symptom trajectories. The longitudinal design enabled the examination of PRO trajectories over time, providing insights that are not possible with cross-sectional studies. Furthermore, this study leveraged real-world data from routine clinical care, unlike many prior studies based on clinical trials or registries, and examined multiple PRO domains across diverse cancer types, offering a comprehensive view of patient experiences. However, there are limitations to consider. The study’s observational nature precludes causal inferences, and the variability in assessment intervals may introduce bias in the trajectory analysis. Additionally, including only seven cancer types and conducting the study at a single institution limit the generalizability of the findings to broader cancer populations. Additionally, participation in the MWC questionnaire was optional, introducing the potential for self-selection bias, which is compounded by the attrition of participants over time. This missing data could lead to biased estimates by reflecting the experiences of a subset of patients who not only chose to participate initially but also remained engaged, thereby further limiting generalizability. Future research should aim to include a broader range of cancer types and explore the impact of specific treatments on PRO trajectories. While our modeling provides valuable insights into average trends and associations, we acknowledge that these aggregate findings may not fully capture the heterogeneity observed at the individual level. Individual patient experiences with cancer and its treatment are highly variable, and their specific PRO trajectories may diverge significantly from population averages. Nevertheless, this data provides crucial information for identifying broad predictors of PROs and generating hypotheses for future research aimed at understanding more individualized responses.

Future research should develop targeted interventions for cancer types with higher symptom burden and explore the underlying biological, psychological, and social mechanisms driving the differences. Incorporating patient preferences and values into care planning will be crucial for enhancing patient-centered oncology care.

## Conclusions

This study highlights the significant variability in PRO trajectories across different cancer types, emphasizing the need for personalized and targeted interventions. Identifying patients at higher risk for poor PRO outcomes enables tailored support, ultimately improving their quality of life and treatment experience.

## Supplementary Material

Supplementary Files

This is a list of supplementary files associated with this preprint. Click to download.

• SupplementaryTables1.pdf

## Figures and Tables

**Table 1:** Patient Characteristics at Baseline

	Total (N = 3809)		Breast (N = 401)		GYN (N = 227)		GI (N = 766)		Head and Neck (N = 412)		Hematologic (N = 991)		Lung (N = 334)		MGS (N = 678)		Pairwise Comparison with Significant Difference
	Mean or N	SE or %	Mean or N	SE or %	Mean or N	SE or %	Mean or N	SE or %	Mean or N	SE or %	Mean or N	SE or %	Mean or N	SE or %	Mean or N	SE or %	
**Age at baseline**	64.1	0.2	60.9	0.6	60.1	0.85	63.1	0.43	65.6	0.54	62.6	0.45	66.8	0.58	68.6	0.41	3,5,6,8,10,11,14,15,16,18,19,20
**Female**	1734	45.4	401	98.3	227	100	335	43.7	129	31.3	452	45.6	190	56.9	.	.	12,14,16,17,19
**Married**	2461	64.5	227	55.6	114	50.2	472	61.6	279	67.7	641	64.7	209	62.6	519	76.5	1,2,3,4,5,6,7,8,9,10,11,12,13,15,17,18,20,21
**Insured**	3758	98.5	403	98.8	224	98.7	751	98	407	98.8	975	98.4	330	98.8	668	98.5	
**Race/Ethnicity**																	
Non-Hispanic White	1625	42.6	166	40.7	74	32.6	303	39.6	216	52.4	426	43	149	44.6	291	42.9	1,3,5,7,8,9,10,11,12,13,14,15,16,17,18
Non-Hispanic Black	307	8.05	43	10.5	24	10.6	45	5.87	22	5.34	94	9.49	16	4.79	63	9.29	2,3,5,7,8,10,13,15,16,18, 19,21
Non-Hispanic Other	97	2.54	15	3.68	7	3.08	19	2.48	11	2.67	19	1.92	14	4.19	12	1.77	2,4,6,11,14,17,19,21
Hispanic	1617	42.4	170	41.7	110	48.5	358	46.7	148	35.9	407	41.1	143	42.8	281	41.4	1,2,3,8,9,10,11,12,13,14, 15,16,17,18
Missing	170	4.45	14	3.43	12	5.29	41	5.35	15	3.64	45	4.54	12	3.59	31	4.57	
**Charlson Comorbidity Index**	6.74	0.06	8.58	0.14	7.43	0.22	8.12	0.12	7.3	0.17	4.47	0.1	8.69	0.18	5.85	0.14	1,3,4,6,9,10,11,12,13,15, 16,17,18,19,20,21
**Cancer Site**																	
Breast	401	10.7	401	100	.	.	.	.	.	.	.	.	.	.	.	.	
Female Genital System	766	20.1	.	.	227	100			.	.	.	.	.	.	.	.	
Gastrointestinal	227	5.95	.	.			766	100	.	.	.	.	.	.	.	.	
Head and Neck	412	10.8	.	.	.	.	.	.	412	100	.	.	.	.	.	.	
Hematologic	991	26	.	.	.	.	.	.	.	.	991	100	.	.	.	.	
Lung	334	8.75	.	.	.	.	.	.	.	.	.	.	334	100	.	.	
Male Genital System	678	17.8	.	.	.	.	.	.	.	.	.	.	.	.	678	100	
**Years since diagnosis**	3.46	0.05	4.27	0.18	3.48	0.22	2.59	0.1	3.51	0.16	3.71	0.11	2.82	0.16	3.87	0.14	2,5,7,12,13,15,19,21
**Metastatic disease**	815	21.4	289	70.8	26	11.5	191	24.9	65	16	12	1.2	91	27.2	141	20.8	1,2,3,4,5,6,7,8,9,10,11,12,13,15,16,17,18,19,20,21
**Treatment Type**																	
Chemotherapy	1033	27.1	143	35	76	33.5	275	35.9	50	12.1	342	34.5	100	29.9	47	6.93	3,5,6,8,11,12,14,15,16,17,18,19,20,21
Immunotherapy	196	5.14	13	3.19	14	6.17	67	8.75	12	2.91	12	1.21	71	21.3	7	1.03	1,2,4,5,6,7,8,9,10,11,12, 13,14,15,16,17,18,19,21
Hormonal Therapy	282	7.39	91	22.3	6	2.64	5	0.65	4	0.97	19	1.92	6	1.8	151	22.3	1,2,3,4,5,7,8,11,13,14,15, 16,18,20,21
Surgery	207	5.42	19	4.66	9	3.96	26	3.39	51	12.4	13	1.31	24	7.19	65	9.59	3,4,5,6,8,9,10,11,12,13,14,15,16,17,18,19,20,21
Radiation	80	2.1	7	1.72	2	0.88	17	2.22	20	4.85	11	1.11	10	2.99	13	1.92	3,5,7,8,10,12,13,17,18,19
**Patient Reported Outcome**																	
Anxiety	51.2	0.16	52.8	0.43	53.1	0.65	51.4	0.35	51	0.49	51.4	0.3	52.3	0.55	48.8	0.37	6,11,15,18,20,21
Depression	46.2	0.16	47.9	0.47	47.7	0.66	46.3	0.34	45.9	0.48	46	0.31	47.4	0.57	44.7	0.36	6,11,21
Pain Interference	51	0.18	52.1	0.52	54	0.77	50.6	0.4	51.5	0.56	51	0.34	51.8	0.63	49.4	0.4	6,7,9,11
Fatigue	48.3	0.19	49.8	0.55	51	0.76	48.2	0.41	47.6	0.59	49.4	0.37	48.7	0.62	45.3	0.42	6,8,11,15,20,21
Physical Function	45.1	0.18	44.1	0.5	42.5	0.71	44.5	0.41	45.7	0.58	45	0.37	43.4	0.61	47.6	0.43	6,11,15,20,21
Health-Related Quality of Life	19.5	0.1	19.1	0.29	18.1	0.43	19.3	0.22	19.3	0.31	19.4	0.19	18.9	0.36	20.9	0.2	6,11,15,18,20,21

GI; gastrointestinal cancer, GYN; gynecological cancer, HRQOL; health-related quality of life, MGS: Male genital system cancer, SE; standard error. Statistically significant *p* values (Bonferroni-adjusted p < .0083): 1 = Breast vs. GYN, 2 = Breast vs. GI, 3 = Breast vs. Head and neck, 4 = Breast vs. Hematologic, 5 = Breast vs. Lung, 6 = Breast vs. MGS, 7 = GYN vs. GI, 8 = GYN vs. Head and neck, 9 = GYN vs. Hematologic, 10 = GYN vs. Lung, 11 = GYN vs. MGS, 12 = GI vs. Head and neck, 13 = GI vs. Hematologic, 14=GI vs. Lung, 15 = GI vs. MGS, 16 = Head and neck vs. Hematologic, 17 = Head and neck vs. Lung, 18 = Head and neck vs. MGS, 19 = Hematologic malignancy vs. Lung, 20 = Hematologic malignancy vs. MGS, 21 = Lung vs. MGS

**Table 2:** Baseline Patient-Reported Outcome Severity Levels by Cancer Type

	Breast (N=401)	GYN (N = 227)	GI (N = 766)	Head and Neck (N = 412)	Hematologic (N = 991)	Lung (N = 334)	MGS (N = 678)	p value^1^
**Anxiety**								**< 0.001**
Normal	242 (61.7%)	121 (54.5%)	462 (62.2%)	257 (64.6%)	600 (62.6%)	195 (61.1%)	488 (73.9%)	
Mild	64 (16.3%)	41 (18.5%)	123 (16.6%)	67 (16.8%)	180 (18.8%)	47 (14.7%)	77 (11.7%)	
Moderate	77 (19.6%)	54 (24.3%)	145 (19.5%)	64 (16.1%)	159 (16.6%)	69 (21.6%)	86 (13.0%)	
Severe	9 (2.3%)	6 (2.7%)	13 (1.7%)	10 (2.5%)	19 (2.0%)	8 (2.5%)	9 (1.4%)	
Missing	9	5	23	14	33	15	18	
**Depression**								0.020
Normal	283 (77.1%)	156 (75.7%)	577 (81.8%)	311 (81.8%)	723 (81.4%)	225 (75.8%)	535 (85.3%)	
Mild	49 (13.4%)	28 (13.6%)	70 (9.9%)	42 (11.1%)	105 (11.8%)	39 (13.1%)	67 (10.7%)	
Moderate	31 (8.4%)	20 (9.7%)	55 (7.8%)	25 (6.6%)	54 (6.1%)	29 (9.8%)	23 (3.7%)	
Severe	4 (1.1%)	2 (1.0%)	3 (0.4%)	2 (0.5%)	6 (0.7%)	4 (1.3%)	2 (0.3%)	
Missing	34	21	61	32	103	37	51	
**Pain Interference**								**0.002**
Normal	207 (54.9%)	104 (50.0%)	430 (60.5%)	224 (58.9%)	538 (59.1%)	170 (56.1%)	409 (64.5%)	
Mild	83 (22.0%)	42 (20.2%)	131 (18.4%)	57 (15.0%)	165 (18.1%)	56 (18.5%)	116 (18.3%)	
Moderate	73 (19.4%)	45 (21.6%)	118 (16.6%)	76 (20.0%)	177 (19.4%)	60 (19.8%)	94 (14.8%)	
Severe	14 (3.7%)	17 (8.2%)	32 (4.5%)	23 (6.1%)	31 (3.4%)	17 (5.6%)	15 (2.4%)	
Missing	24	19	55	32	80	31	44	
**Fatigue**								**< 0.001**
Normal	249 (68.6%)	124 (62.3%)	491 (71.3%)	278 (74.3%)	600 (68.0%)	212 (71.6%)	505 (80.9%)	
Mild	55 (15.2%)	32 (16.1%)	106 (15.4%)	35 (9.4%)	121 (13.7%)	42 (14.2%)	71 (11.4%)	
Moderate	49 (13.5%)	35 (17.6%)	74 (10.7%)	47 (12.6%)	136 (15.4%)	31 (10.5%)	42 (6.7%)	
Severe	10 (2.8%)	8 (4.0%)	18 (2.6%)	14 (3.7%)	25 (2.8%)	11 (3.7%)	6 (1.0%)	
Missing	38	28	77	38	109	38	54	
**Physical Function**								**< 0.001**
Normal	39 (10.7%)	19 (9.5%)	89 (13.1%)	63 (17.1%)	143 (16.1%)	32 (10.7%)	128 (21.3%)	
Mild	199 (54.8%)	95 (47.5%)	347 (51.2%)	185 (50.1%)	423 (47.5%)	145 (48.7%)	323 (53.7%)	
Moderate	90 (24.8%)	62 (31.0%)	176 (26.0%)	83 (22.5%)	255 (28.7%)	92 (30.9%)	123 (20.5%)	
Severe	35 (9.6%)	24 (12.0%)	66 (9.7%)	38 (10.3%)	69 (7.8%)	29 (9.7%)	27 (4.5%)	
Missing	38	27	88	43	101	36	77	
**HRQOL**								**< 0.001**
Low	104 (31.6%)	66 (36.3%)	187 (29.3%)	115 (32.5%)	258 (30.8%)	88 (33.2%)	114 (19.9%)	
Normal/High	225 (68.4%)	116 (63.7%)	451 (70.7%)	239 (67.5%)	580 (69.2%)	177 (66.8%)	460 (80.1%)	
Missing	72	45	128	58	153	69	104	

GI; gastrointestinal cancer, GYN; gynecological cancer, HRQOL; health-related quality of life, MGS: Male genital system cancer.

1 Pearson’s Chi-squared test

**Table 3:** Factors Associated with Patient-Reported Outcomes

	Anxiety		Depression		Pain Interference		Fatigue		Physical Function		HRQOL	
	Estimate	SE	Estimate	SE	Estimate	SE	Estimate	SE	Estimate	SE	Estimate	SE
**Intercept**	51.68*	1.50	46.96*	1.47	46.35*	1.57	44.24*	1.72	63.47*	1.59	21.08*	0.88
**Time**	−0.19*	0.04	−0.01	0.04	−0.11	0.04	0.06	0.04	0.04	0.04	0.01	0.02
**Age at baseline**	−0.03	0.01	0.00	0.01	0.01	0.01	0.01	0.01	−0.20*	0.01	0.00	0.01
**Partnered vs. unpartnered**	−1.44*	0.30	−1.67*	0.29	−1.22*	0.31	−1.46*	0.34	1.29*	0.31	0.94*	0.17
**Insured vs. Uninsured**	−0.66	1.15	−1.61	1.12	0.44	1.20	0.39	1.32	0.62	1.21	−0.08	0.67
**Charlson Comorbidity Index**	0.17*	0.05	0.21*	0.04	0.38*	0.05	0.42*	0.05	−0.55*	0.05	−0.24*	0.03
**Metastatic disease**	0.73	0.41	0.61	0.40	0.26	0.43	0.77	0.47	−0.28	0.43	−0.37	0.24
**Years since diagnosis**	−0.10	0.04	−0.09	0.04	−0.08	0.05	−0.10	0.05	0.14*	0.05	0.10*	0.03
**Race/Ethnicity**												
Hispanic	0.16	0.30	−0.55	0.30	0.06	0.32	−2.35*	0.35	−0.38	0.32	0.39	0.18
Non-Hispanic Black	−2.43*	0.53	−1.92*	0.52	1.22	0.55	−1.55	0.61	0.20	0.56	0.16	0.31
Non-Hispanic Other	−0.88	0.88	−0.22	0.86	0.06	0.92	−1.18	1.00	−0.72	0.92	0.54	0.51
Non-Hispanic White	0.00	.	0.00	.	0.00	.	0.00	.	0.00	.	0.00	.
**Cancer Site**												
Breast	2.61*	0.63	1.66*	0.62	1.27	0.67	2.28*	0.72	−2.82*	0.68	−0.70	0.38
GYN	3.60*	0.74	2.79*	0.73	3.59*	0.80	4.43*	0.86	−5.56*	0.82	−1.98*	0.45
GI	1.52*	0.54	0.83	0.53	0.48	0.58	2.09*	0.61	−2.78*	0.58	−0.91*	0.32
Head and Neck	1.36	0.60	0.72	0.59	1.37	0.65	1.53	0.69	−2.01*	0.65	−1.13*	0.36
Hematologic	2.31*	0.50	1.37*	0.49	2.22*	0.54	4.10*	0.58	−4.31*	0.55	−1.52*	0.30
Lung	2.48*	0.66	1.39	0.65	0.95	0.71	1.63	0.76	−2.57*	0.72	−0.89	0.40
MGS	0.00	.	0.00	.	0.00	.	0.00	.	0.00	.	0.00	.
**Chemotherapy**	0.11	0.34	0.19	0.33	0.91*	0.35	2.28*	0.39	−2.53*	0.36	−1.14*	0.20
**Immunotherapy**	−0.46	0.67	0.19	0.65	0.18	0.70	−0.14	0.76	0.15	0.70	0.09	0.39
**Hormonal Therapy**	−0.24	0.58	0.01	0.56	−0.02	0.60	0.65	0.66	−0.72	0.61	−0.13	0.34
**Surgery**	0.11	0.63	−0.06	0.62	2.22*	0.66	0.14	0.72	−2.23*	0.67	−0.18	0.37
**Radiation**	−1.66	0.99	−1.34	0.98	1.14	1.04	−0.03	1.15	0.19	1.05	−0.13	0.58
**Interaction**												
Time*Breast	0.06	0.07	−0.08	0.06	0.15	0.07	−0.08	0.07	−0.08	0.07	−0.01	0.04
Time*GYN	0.00	0.09	−0.08	0.08	0.01	0.09	−0.04	0.09	−0.04	0.09	0.00	0.05
Time*GI	0.06	0.06	0.02	0.05	0.10	0.06	−0.02	0.06	−0.07	0.06	−0.04	0.03
Time*Head and Neck	−0.02	0.06	−0.05	0.06	−0.05	0.07	−0.24*	0.07	0.12	0.06	0.08*	0.03
Time*Hematologic	−0.03	0.05	−0.04	0.05	0.06	0.05	−0.17*	0.06	0.04	0.05	0.02	0.03
Time*Lung	0.02	0.07	0.06	0.07	0.05	0.07	0.10	0.08	−0.16	0.07	−0.04	0.03
Time*MGS	0.00	.	0.00	.	0.0	.	0.00	.	0.00	.	0.00	.

GI; gastrointestinal cancer, GYN; gynecological cancer, HRQOL; health-related quality of life, MGS: Male genital system cancer, SE; standard error. Reference groups: cancer site: male genital system; marital status: unpartnered; insurance status: uninsured; metastatic disease: no; chemotherapy: no; immunotherapy: no; hormonal therapy: no; surgery: no; radiation: no; interaction term: time*male genital system.

* = Statistically significant *p* values (Bonferroni-adjusted p < .0083).

1a) PROMIS Anxiety T-score 1b) PROMIS Depression T-score

1c) PROMIS Pain Interference T-score 1d) PROMIS Fatigue T-score

1e) PROMIS Physical Function T-score 1f) FACT-G7 score (Health-related quality of life)

## Data Availability

The datasets generated during and/or analysed during the current study are available from the corresponding author on reasonable request.
